# Exploring the Causal Relationship Between Arterial and Venous Thromboembolism: A Case Series With Review of Literature

**DOI:** 10.7759/cureus.37660

**Published:** 2023-04-16

**Authors:** Prakash Upreti, Sudharsan R Gongati, Neelanjana Pandey, Muhammad Saad, Timothy Vittorio

**Affiliations:** 1 Internal Medicine, Rochester Regional Health, Rochester, USA; 2 Internal Medicine, BronxCare Health System, Bronx, USA; 3 Cardiology, BronxCare Health System Affiliated with the Icahn School of Medicine at Mount Sinai, Bronx, USA; 4 Cardiovascular Disease, BronxCare Health System, Bronx, USA

**Keywords:** arterial thrombosis, deep vein thrombosis (dvt), pulmonary embolism (pe), acute coronary syndrome, venous thromboembolism

## Abstract

Venous thromboembolism (VTE) occurs due to venous stasis or low flow state within the blood vessels, resulting in subsequent fibrin and platelet aggregation leading to thrombosis. Arterial thrombosis affects various arteries including coronaries and is primarily due to platelet aggregation with little fibrin deposition leading to thrombosis. Although both arterial and venous thrombosis are considered as separate entities, some studies have suggested an association between them despite having distinctive causative factors. We retrospectively reviewed patients at our institution who were admitted with acute coronary syndrome (ACS) and underwent cardiac catheterization over a decade between 2009 and 2020 to look for patients who had both venous thromboembolic events and ACS. Here, we report a case series of three such patients who were found to have both VTE and coronary arterial thrombosis. However, it is unclear if having one of venous vs arterial clot increases the risk of having other vascular conditions, and further studies are needed to evaluate this hypothesis in the near future.

## Introduction

Historically, arterial and venous thromboembolism (VTE) are regarded as separate entities due to the nature of the disease involving arteries and veins, respectively. Whereas arterial thrombosis is due to platelet aggregation with little fibrin deposits, venous thrombosis occurs due to stasis or low flow state, fibrin, and platelet aggregation as per Virchow's triad. A few studies have found a connection between these two vascular entities, and this connection may be caused by the fact that both diseases share some risk factors even though they each have unique pathophysiology.

Acute coronary syndrome (ACS) refers to a group of conditions that include ST elevation myocardial infarction (STEMI), non-ST elevation myocardial infarction (NSTEMI), and unstable angina (UA) [1]. Whereas ACS signifies coronary arterial thrombosis, epidemiologic studies have suggested an association with other systemic thromboembolic events including deep vein thrombosis (DVT), pulmonary embolism (PE), and stroke.

## Case presentation

We conducted a retrospective chart review of adult patients above 18 years admitted with ACS who underwent cardiac catheterization at BronxCare Hospital Center, Bronx, NY, from July 2009 until August 2020. The clinical data including patient demographic characteristics, comorbid conditions, reason for admission, length of hospital stay, days on mechanical ventilation, clinical outcomes, laboratory results, and other pertinent clinical information of the patients who met the above criteria were collected with the help of an electronic medical record system. Patients with a prior history of arterial or VTE (peripheral artery disease, coronary artery disease, stroke, DVT, and PE) were excluded.

During the timeline as indicated above, we found three patients who were noted to have concomitant VTE and coronary arterial thrombosis. Two of the patients had both coronary arterial thrombosis and VTE during the same admission, and the other patient had coronary arterial thrombosis in the index admission and venous thrombosis in subsequent admission. Here, we report a case series of three such patients who were found to have both VTE and coronary arterial thrombosis.

Case 1

**Figure 1:**
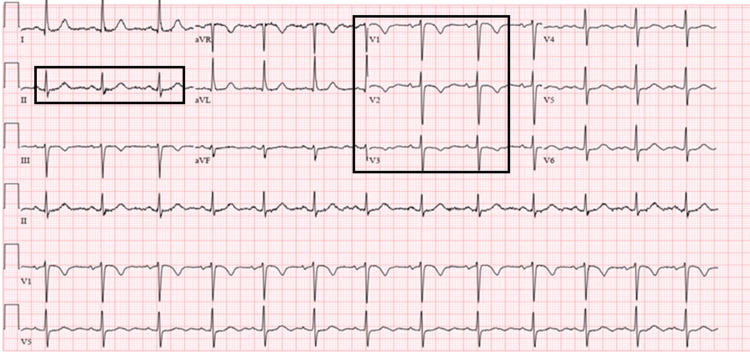
ECG showing normal sinus rhythm, a ventricular rate of 87 bpm, ST depression in lead II, and T wave inversion in leads V1-V3.

A 50-year-old Hispanic female with a medical history of hypertension, human immunodeficiency virus disease on antiretroviral medications, dyslipidemia, and gastroesophageal reflux disease presented to the emergency room with a complaint of chest pain that started on the day of presentation while descending the stairs. The chest pain was substernal, constant, squeezing, radiating to the back, and associated with diaphoresis and clammy sensation. The patient was a former smoker and quit smoking 10 years ago. The vital signs on presentation were a blood pressure of 163/90 mmHg, a heart rate of 95 beats per minute, a respiratory rate of 16 breaths per minute, and 97% oxygen saturation on room air. Physical examination otherwise was unremarkable. Electrocardiogram (ECG) showed T wave inversion (TWI) in leads V1 and V2 (Figure 1).

The initial troponin I level was within normal limits; however, the repeated level after six hours was elevated. Repeat ECG showed ST segment depression in lead II and more pronounced TWI in leads V1-V3. The patient received aspirin 325 mg with improvement in pain and was admitted to the coronary care unit (CCU) with the diagnosis of NSTEMI. The patient received a loading dose of clopidogrel and was started on therapeutic anticoagulation with a heparin drip. Subsequent lab results showed decreasing troponin levels (0.087 > 0.687 ng/mL). The patient’s urine toxicology was negative and the chest X-ray was normal. 

**Figure 2:**
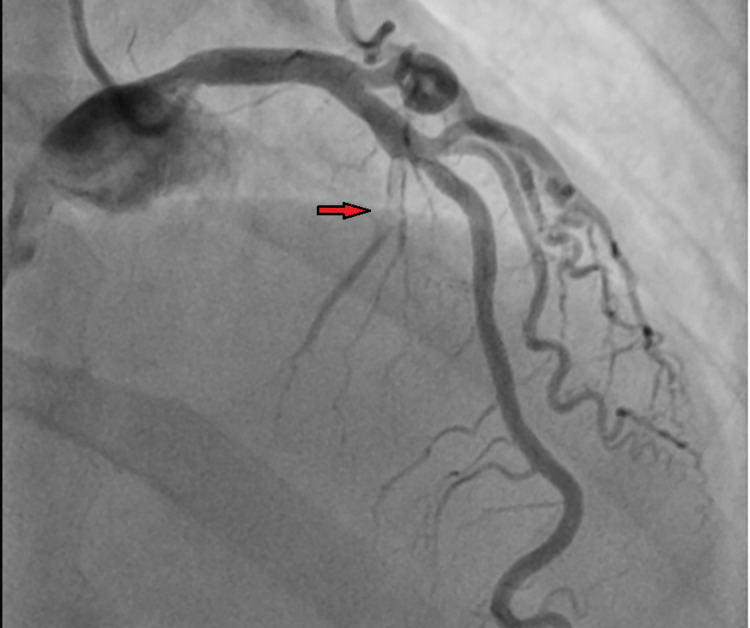
Coronary angiogram showing 100% stenosis of the first septal branch of the left anterior descending artery (red arrow).

The patient underwent cardiac catheterization, which showed 100% stenosis of the first septal branch of the left anterior descending (LAD) artery and 50% stenosis of the proximal right coronary artery (Figure 2).

**Figure 3:**
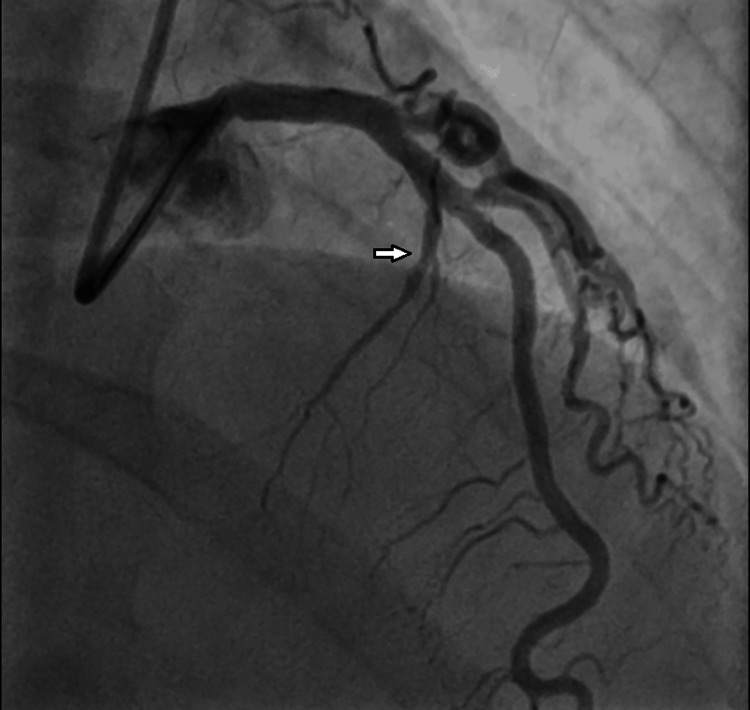
Coronary angiogram showing a good flow of the first septal branch of the left anterior descending artery post-thrombectomy. White arrow showing post-TIMI grade III flow of the first septal branch of the left anterior descending artery post-thrombectomy.

The patient underwent successful aspiration thrombectomy and percutaneous old balloon angioplasty (POBA) of the first septal branch with a resolution of the thrombus (Figure 3).

**Figure 4:**
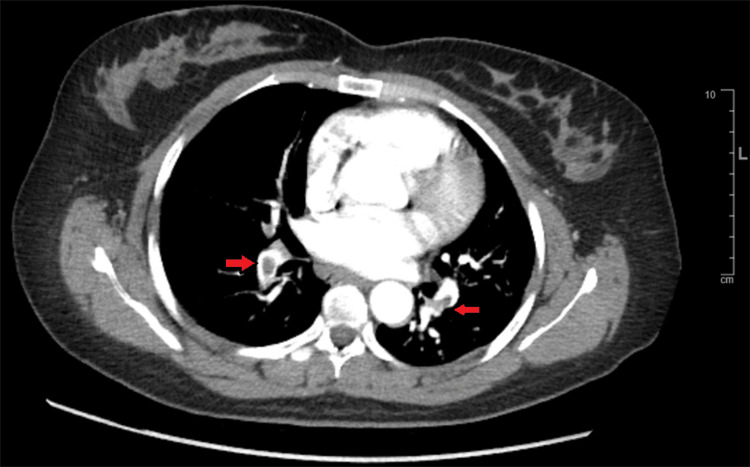
CT scan of the chest showing bilateral pulmonary embolism (red arrows).

Additionally, small distal embolization was noted at the very terminal part of LAD, which was treated with wire dottering. Echocardiogram showed a left ventricular ejection fraction (LVEF) of 56% with hypokinesis of the basal anteroseptal segment. A contrast CT scan of the chest ruled out aortic dissection but showed bilateral PE (Figure 4).

**Figure 5:**
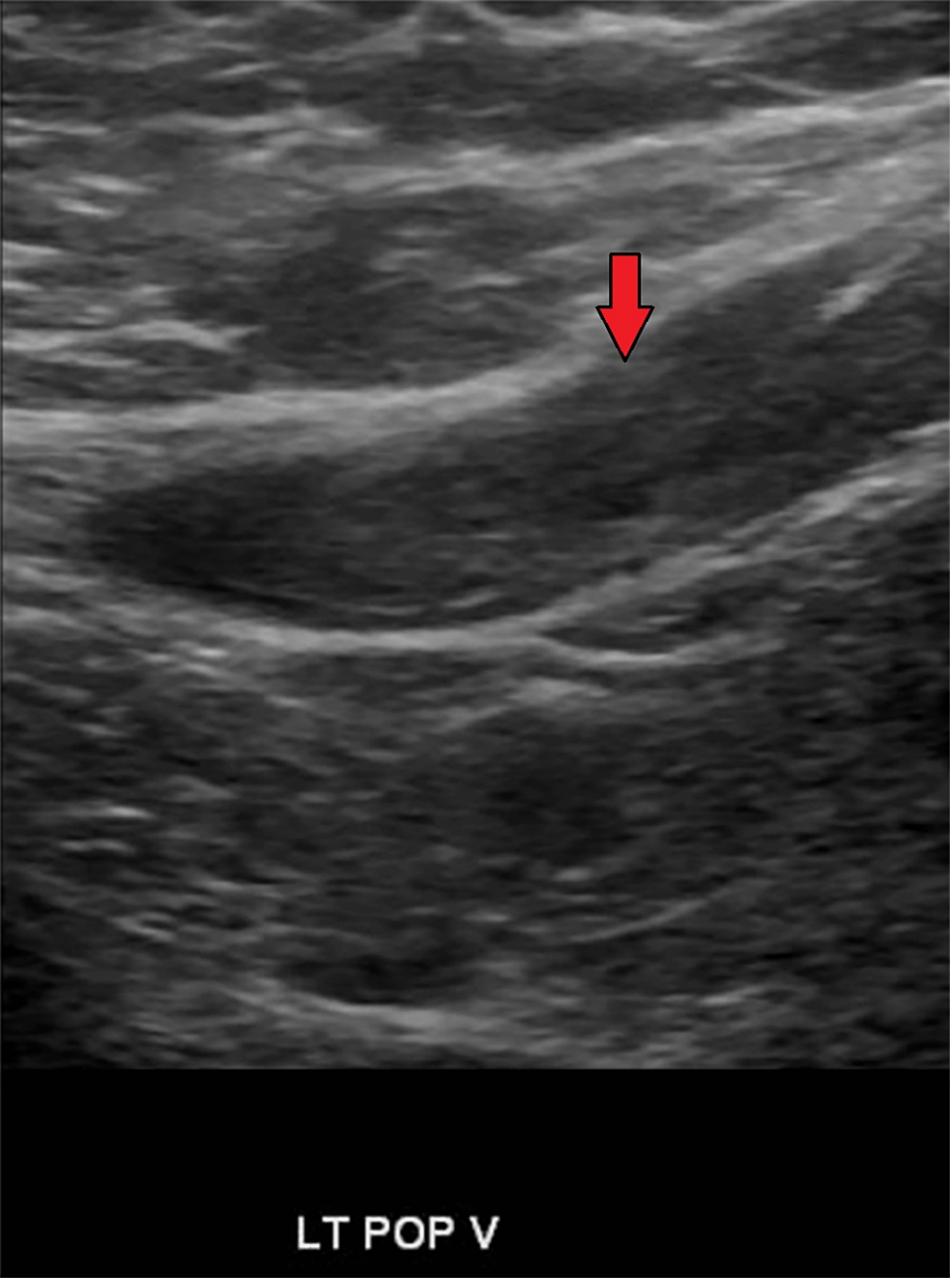
Deep vein thrombosis of the left popliteal vein (red arrow).

Ultrasound of lower extremities was also done, which showed DVT in the left popliteal vein (Figure 5).

Hypercoagulability workup was unremarkable. The patient was started on warfarin with enoxaparin bridging and was later discharged on warfarin with cardiology outpatient follow-up. 

Case 2

A 50-year-old African American male with a medical history of hypertension for 10 years on amlodipine and dyslipidemia for two years on atorvastatin presented to the emergency room with a complaint of chest pain. He described chest pain as midsternal and pressure-like while on his way to work, and it was associated with lightheadedness, nausea, and diaphoresis. The vital signs on presentation were a blood pressure of 150/84 mmHg, a heart rate of 70 beats per minute, a respiratory rate of 24 breaths per minute, and 99% oxygen saturation at 2 l/min of oxygen via nasal cannula. Physical examination was otherwise unremarkable.

**Figure 6:**
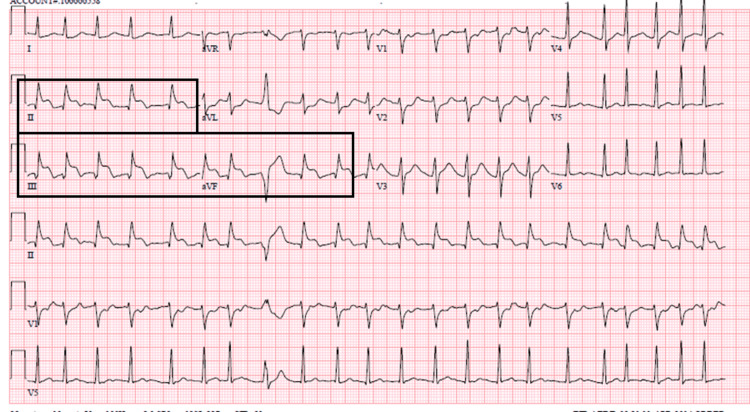
ECG showing ST elevation in inferior leads with atrial fibrillation.

ECG was consistent with atrial fibrillation (AF) and ST segment elevation in inferior leads with reciprocal changes (Figure 6).

**Figure 7:**
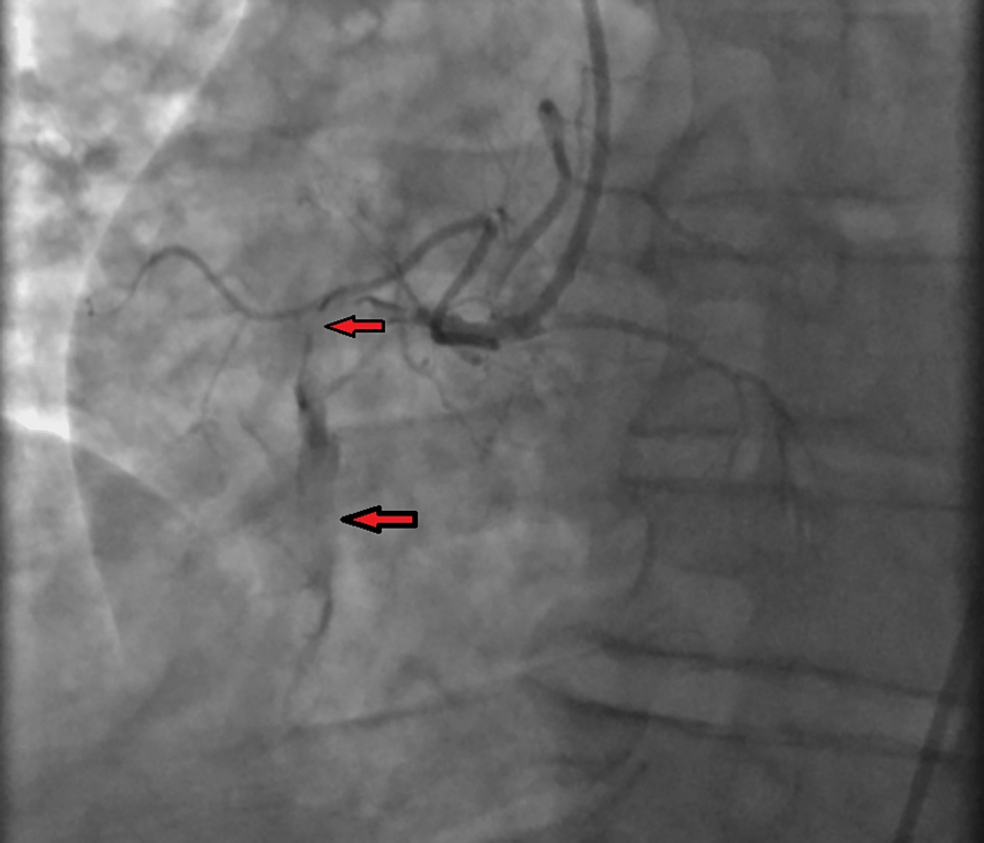
Coronary angiogram showing 100% stenosis of the proximal and mid-right coronary artery (red arrows).

The patient was loaded with aspirin, clopidogrel, and heparin bolus. He immediately underwent cardiac catheterization and coronary angiogram, which showed 100% stenosis of RCA (Figure 7), and he underwent successful percutaneous coronary intervention (PCI) of the proximal and mid-RCA.

However, the patient developed ventricular fibrillation (VF) intermittently and received six shocks, atropine, and amiodarone as per ACLS protocol. The patient went into asystole, and cardiopulmonary resuscitation was initiated with the return of spontaneous circulation achieved in five minutes. Intra-aortic balloon pump was inserted as the patient was in cardiogenic shock, and he was started on a dobutamine drip and an amiodarone drip for VF and was transferred to another facility for possible extracorporeal membrane oxygenation (ECMO).

**Figure 8:**
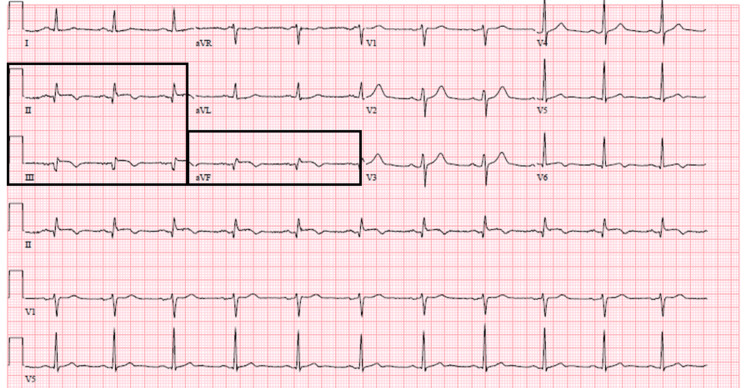
ECG showing ST elevation in inferior leads.

Two months later, the patient had acute onset of chest pain, and he was brought to the emergency room as a STEMI alert. ECG was consistent with ST elevations in inferior leads (Figure 8).

He was non-compliant with aspirin and clopidogrel. Cardiac catheterization showed 40% stenosis of the mid-LAD and 100% in-stent restenosis of the proximal RCA. Guidewire could not be passed to the mid-RCA due to heavy thrombus burden. A proximal RCA balloon angioplasty was done, and TIMI II flow was achieved; however, the patient had VF during the procedure and received DC cardioversion. Later, the patient was started on an amiodarone drip and subsequently transferred to a tertiary care center where he underwent automatic implantable cardioverter-defibrillator (AICD) placement.

**Figure 9:**
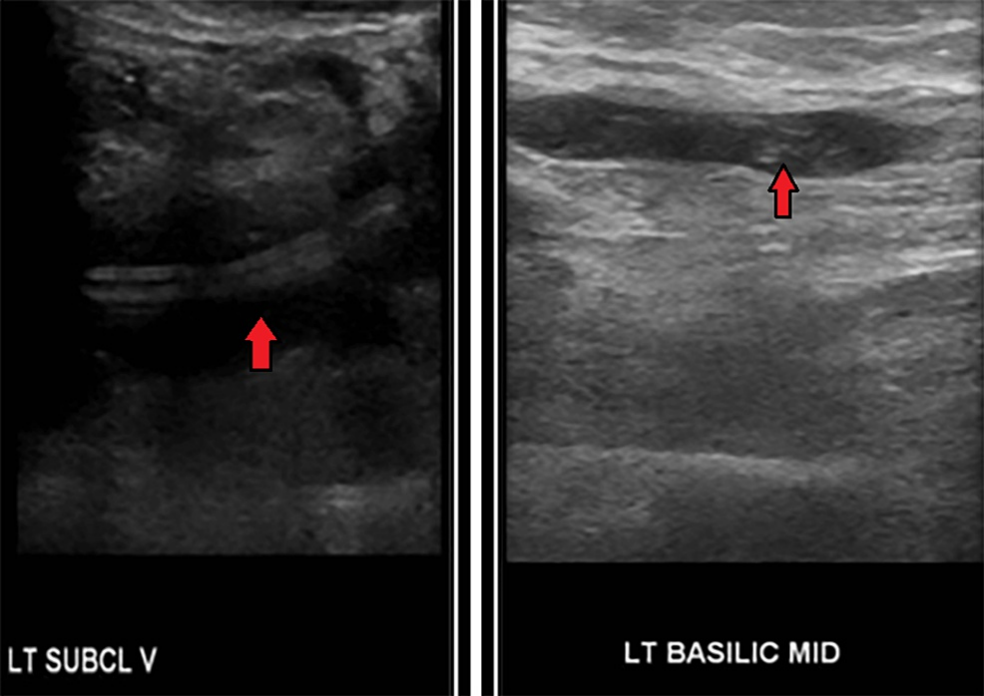
Deep vein thrombosis of the left subclavian vein and left basilic vein (red arrows).

A week later, the patient presented to the emergency room with complaints of left arm swelling since the past one day. He was admitted with suspicion of provoked DVT since he had a recent AICD placement. A heparin drip was initiated. Ultrasound of the left upper extremity showed DVT of left subclavian, axillary, and basilic veins (Figure 9).

Cardiac catheterization this time showed double vessel disease with a patent stent in the proximal RCA with an occluded stent in the mid-RCA and grade II collateral flow from the LAD to distal RCA. He was discharged on warfarin, and hypercoagulability workup was significant for the elevated homocysteine level.

Case 3

An 84-year-old African American female with a medical history of hypertension and urinary incontinence presented to the emergency room with a complaint of sudden onset chest pain while using the restroom. She might have had vagal stimulation from a bowel movement, which could have been contributory to this presentation. She described the pain as substernal heaviness, 10/10 in intensity, non-radiating, associated with diaphoresis, lasted about an hour, and resolved spontaneously. The patient reported no active chest pain on presentation to the emergency room. She was an active smoker with a 25-pack-year history of smoking.

**Figure 10:**
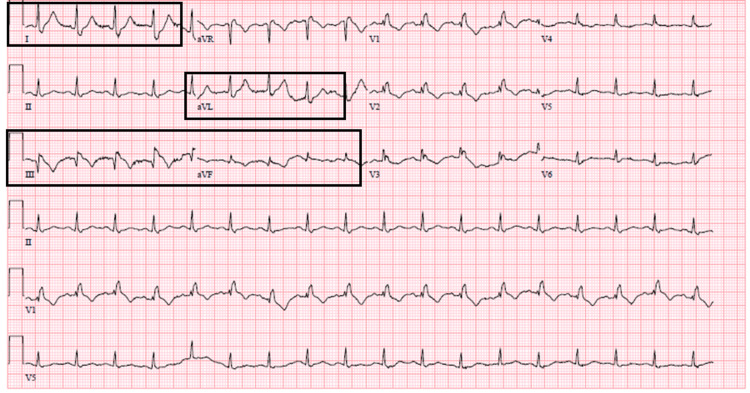
ECG showing 0.5-1 mm ST segment elevation in lead III, TWI in leads III and aVF, and minimal ST depression in leads I and aVL. TWI, T wave inversion

She had a blood pressure of 121/70 mmHg, a heart rate of 110 beats per minute, a respiratory rate of 20 breaths per minute, and 94% oxygen saturation on room air. The physical examination otherwise was significant for pitting edema over both lower extremities. ECG showed sinus rhythm with a ventricular rate of 114 beats per minute, 0.5-1 mm ST segment elevation in lead III, TWIs in leads III and aVF, and minimal ST depression in leads I and aVL (Figure 10).

**Figure 11:**
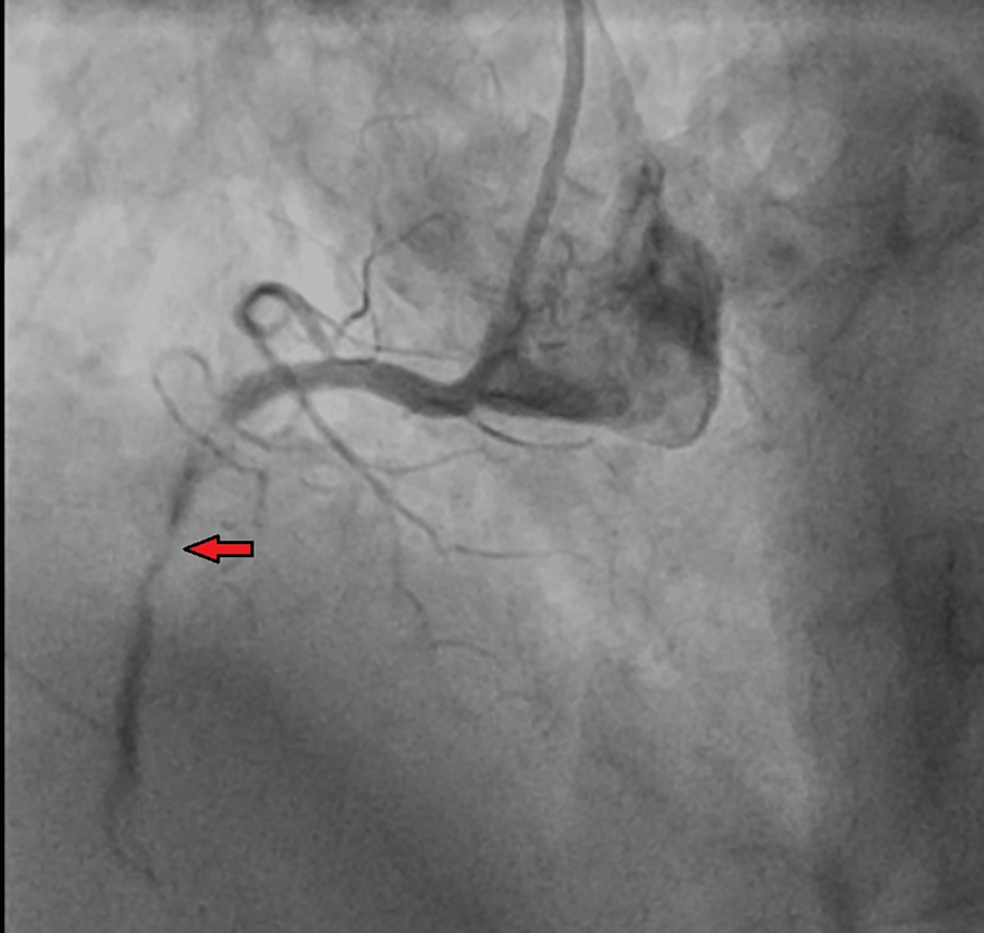
Coronary angiogram showing 100% obstruction of the mid-right coronary artery (red arrow).

High sensitivity troponin T level was 237 (normal, <12 ng/L), and proBNP level was 1711 (normal, <125 pg/ml). The patient was diagnosed with NSTEMI; received aspirin, clopidogrel, and heparin bolus; and underwent cardiac catheterization. Coronary angiogram showed 90% stenosis of the proximal left circumflex artery, 80% stenosis of the first obtuse marginal, 70% stenosis of the distal RCA, and 100% stenosis of the mid-RCA (Figure 11).

**Figure 12:**
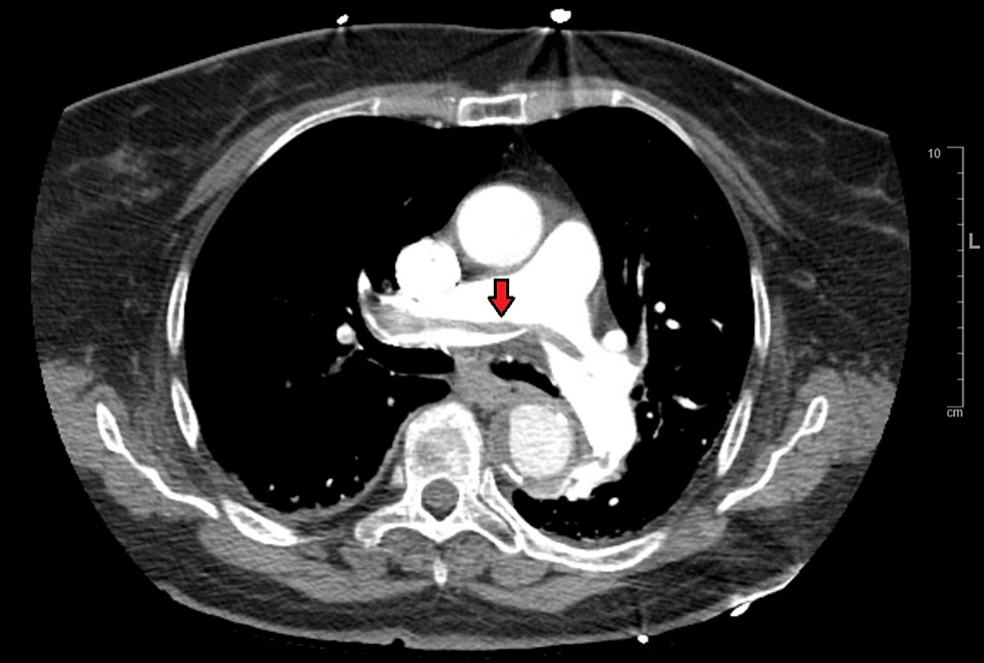
CT chest showing saddle pulmonary embolism (red arrow).

The patient had successful PCI of the mid-RCA with a drug-eluting stent. Echocardiogram showed an LVEF of 55.9%, abnormal septal motion consistent with right ventricular volume and pressure overload, abnormal diastolic filling, and right ventricular free wall hypokinesis with sparing of the apex (McConnell's sign). The patient was empirically started on therapeutic enoxaparin for suspected PE. CT chest with contrast showed saddle pulmonary embolus, large distal right and left main pulmonary arterial emboli with extension to the upper and lower lobe peripheral branches (Figure 12), and evidence of right heart strain.

**Figure 13:**
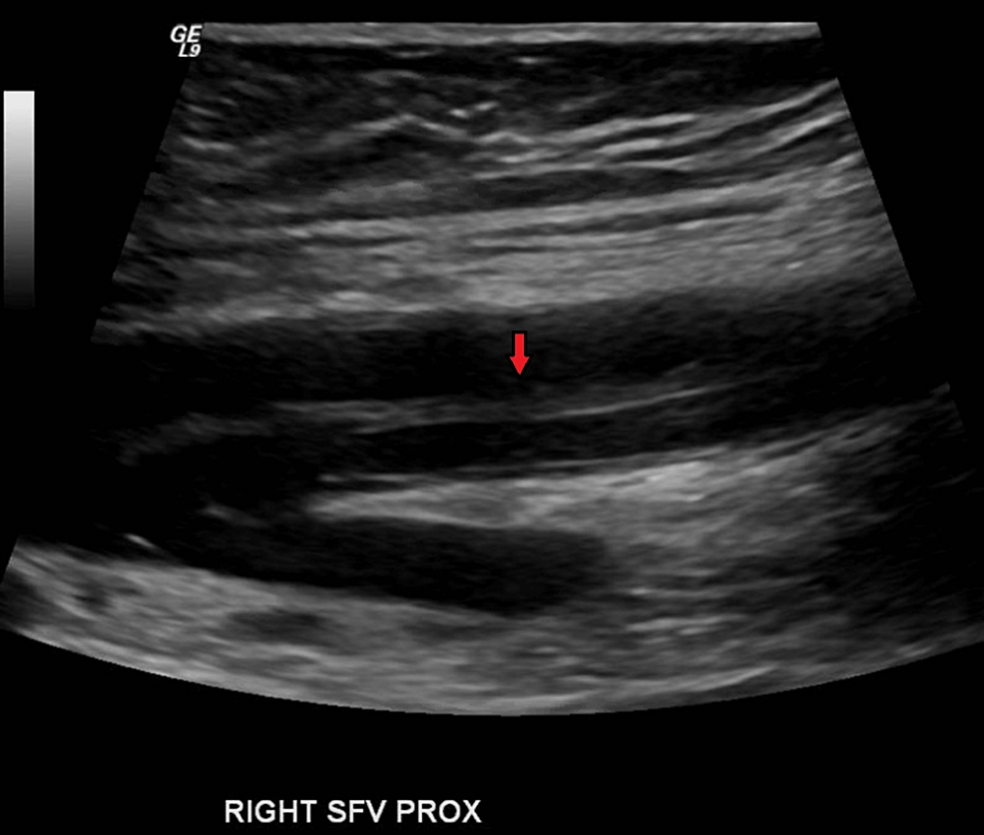
Deep vein thrombosis of the right superficial femoral vein (red arrow).

Ultrasound of the lower extremities showed acute thrombus in right superficial femoral and popliteal veins (Figure 13).

The patient was started on coumadin with enoxaparin bridging during the hospital course and subsequently was discharged on coumadin, aspirin, clopidogrel, and high-intensity statin. Upon follow-up, a month later, coumadin was switched to direct acting oral anticoagulant (apixaban). Hypercoagulability workup was significant for elevated homocysteine levels.

## Discussion

VTE is an umbrella term used to describe blood clot within the vasculature that provides blood return to the heart. It includes DVT and PE. The common causes of VTE include any major surgery, active cancer, trauma, bone fracture, prolonged immobilization, cigarette smoking, and among women, pregnancy or the puerperium, oral contraceptive use, estrogen use, and progestin use. Patients with hypercoagulable or genetic risk factor predisposition are at increased risk.

ACS refers to a group of myocardial ischemic conditions that includes UA, NSTEMI, and STEMI [1]. It occurs due to plaque disruption with superimposed thrombosis [2] in the coronary artery. The risk factors are cigarette smoking, obesity, hypertension, hyperlipidemia, diabetes mellitus, and male sex.

VTE and arterial thrombosis are historically considered as two distinct disease entities with distinct pathologies and etiologies. Venous thrombi mainly consist of red blood cells and fibrin [3], while arterial thrombi mainly consist of platelets [4]. This is the reason why the anticoagulant agent is prescribed in VTE, while anti-platelets are prescribed in arterial thromboembolic diseases. The risk factors for VTE and arterial thrombosis are also distinctly different; however, many patients have shown overlapping risk factors. Acute arterial thrombosis is usually due to atherosclerotic plaque rupture [5,6], while VTE events mainly occur due to a low flow state or venous stasis [3]. As described above, since VTE and arterial thrombosis are different disease lines, we wished to find an associating risk factor that connects the two given the cases of dual presentation.

Prandoni found that in 244,865 patients, cigarette smoking was an independent risk factor for arterial thrombosis and is an already well-established risk factor for VTE [6]. The risk of VTE was higher with more cigarettes smoked per day, according to a meta-analysis of 21 studies that found current smoking to be associated with an elevated risk of the condition (RR, 1.24; 95% CI, 1.14-1.35) as well as previous smoking (RR, 1.05; 95% CI, 1.01-10.10) [7]. VTE risk was 6.2 times higher in people who were obese. Patients over 50 and cases falling into classes II and III of obesity were at the highest risk of VTE related to obesity [8]. Marcucci et al. found that for 603 patients with VTE, there were high levels of lipoprotein(a). Lipoprotein(a) was an independent risk factor for idiopathic VTE (OR, 2.1; 95% CI, 1.4-3.2); meanwhile, it is a known marker for atherosclerosis of the arterial system [9].

For 89 patients with proven VTE (51.7%), coronary artery calcium was found to be more common than age- and gender-matched controls with VTE (28.1%) (OR, 4.3; 95% CI, 1.9-10.1) [10]. Diabetes and hypertension were also found to be statistically significant for findings of VTE-positive status.

Becattini et al. performed a prospective study of 360 patients with first-ever PE. Patients with unprovoked PE appeared to have a greater rate of arterial events as well (RR, 7.2; 95% CI, 1.71-30.45). Index PE is an independent risk factor for future arterial events per the age-controlled data [11]. A prolonged 10-year follow-up (patients were randomized from April 1988 to April 1991 and followed for 10 years) of the DURAC research in patients with VTE [12] corroborated these findings. In this investigation, death from AMI and stroke was greater in patients with previous VTE than in the general population (standardized incidence ratio, 1.28; 95% CI, 1.00-1.56). Prandoni et al. conducted a prospective follow-up study of 1919 patients with a first episode of VTE for any incidence of symptomatic arterial disease. After a median follow-up of 4 years, they found that 15.1% of patients with idiopathic VTE had at least one arterial event, compared to 8.5% of patients with secondary VTE [13].

One study has demonstrated that patients with a history of arterial cardiovascular events were at increased risk of VTE events in the first three months following the index event [14]. On the other hand, a longitudinal cohort study in patients aged 20-39 years of age presenting with unprovoked VTE showed that they had an increased risk of myocardial infarction as compared to controls [15]. Apart from having acute coronary events, patients with prior VTE are also shown to be at an increased risk for hospitalization due to MI, stroke, and transient ischemic attack within a year after the episode of VTE [16,17]. The results of these studies suggest that patients with VTE are at increased risk of subsequent arterial cardiovascular events. Although, studies have not described the pathophysiology of such an occurrence. The underlying mechanism of thrombotic states could be hypercoagulability such as homocysteinemia, lupus anticoagulant, antiphospholipid antibodies, or an intracardiac shunt such as patent foramen ovale or provoked state such as prolonged immobility. This implies that arterial and venous thrombosis may share common mechanisms or risk factors. We can hence conclude that venous and arterial thrombosis are two aspects of the same disease (i.e., thrombosis), which may electively affect genetically predisposed individuals and manifest as either venous thrombotic events or arterial thrombotic events depending on the presence of underlying risk factors.

The key limitations in this case series are the small size and retrospective nature. Also, the patients had risk factors like HIV, AF, and hyperhomocysteinemia. As researchers, we were interested in discovering an association between the simultaneous occurrence of arterial and venous thrombosis.

## Conclusions

After a detailed review of the literature, we can say that there is likely a causal association between venous and arterial thromboembolic events based on the above case series and discussion. However, the amount of evidence to support this possibility is very limited, and further studies are needed to evaluate if any definite association exists between the two entities. This case series highlights the importance of acute coronary arterial thrombosis having a correlation with VTE. Understanding the relationship is crucial because prophylaxis and/or treatment of one condition may benefit patients as well as medical professionals by preventing hospitalizations caused by the other condition, lowering the overall incidence of such events, and lowering the cost of care.
